# The Existence of Periodic Orbits and Invariant Tori for Some 3-Dimensional Quadratic Systems

**DOI:** 10.1155/2014/705703

**Published:** 2014-03-26

**Authors:** Yanan Jiang, Maoan Han, Dongmei Xiao

**Affiliations:** ^1^Department of Mathematics, Shanghai Normal University, Shanghai 200234, China; ^2^Department of Mathematics, Shanghai Jiaotong University, Shanghai 200240, China

## Abstract

We use the normal form theory, averaging method, and integral manifold theorem to study the existence of limit cycles in Lotka-Volterra systems and the existence of invariant tori in quadratic systems in ℝ^3^.

## 1. Introduction

It is well known that *n*-dimensional generalized Lotka-Volterra systems are widely used as the first approximation for a community of *n* interacting species, each of which would exhibit logistic growth in the absence of other species in population dynamics. And this system is of wide interest in different branches of science, such as physics, chemistry, biology, evolutionary game theory, and economics. We refer the reader to the book of Hofbauer and Sigmund [1] for its applications. The existence of limit cycles and invariant tori for these models is interesting and significant in both mathematics and applications since the existence of stable limit cycles and invariant tori provided a satisfactory explanation for those species communities in which populations are observed to oscillate in a rather reproducible periodic manner (cf. [2–4] and references therein).

To study the bifurcation of Lotka-Volterra class, we consider three-dimensional generalized Lotka-Volterra systems

(1) dXi(t)dt=Xi(t)(βi+∑j=13αijXj(t)‍), i=1,2,3,

which describes the interaction of three species in a constant and homogeneous environment, where *X*_*i*_(*t*) is the number of individuals in the *i*th population at time *t* and *X*_*i*_(*t*) ≥ 0, *β*_*i*_ is the intrinsic growth rate of the *i*th population, the *α*_*ij*_ are interaction coefficients measuring the extent to which the *j*th species affects the growth rate of the *i*th, *β*_*i*_ and *α*_*ij*_ are parameters, and the values of these parameters are not very small usually.

Over the last several decades, many researchers have devoted their effort to study the existence and number of isolated periodic solutions for system (1). There have been a series of achievements and unprecedented challenges on the theme even if system (1) is a competitive system (cf. [5–12]). In [13], Bobieński and Żołądek gave four components of center variety in the three-dimensional Lotka-Volterra class and studied the existence and number of isolated periodic solutions by certain Poincaré-Melnikov integrals of a new type. In [14], Llibre and Xiao used the averaging method to study the existence of limit cycles of three-dimensional Lotka-Volterra systems. In this paper, we will use the normal form theory to study the same question. And furthermore, we will give the existence of invariant tori in a system of the form (2).

This paper is organized as follows. In Section 2, we obtain some preliminary theorems about a normal form system of degree two in ℝ^3^ with two small parameters *λ*_1_ and *λ*_2_ and other bounded parameters. In Section 3, we first change the system (1) into a system of the form

(2) $$\begin{array}{c} \frac{dU}{dt}=u\varepsilon U+vV+\underset{i+j+k=2}{\sum }a_{ijk}U^{i}V^{j}W^{k},\\ \frac{dV}{dt}=-vU+u\varepsilon V+\underset{i+j+k=2}{\sum }b_{ijk}U^{i}V^{j}W^{k},\\ \frac{dW}{dt}=\varepsilon W+\underset{i+j+k=2}{\sum }c_{ijk}U^{i}V^{j}W^{k}, \end{array}$$

where *a*_*ijk*_, *b*_*ijk*_, and *c*_*ijk*_ for *i*, *j*, *k* = 0,1, 2 are functions of the parameters *β*_*i*_ and *α*_*ij*_ in system (1), *u* and *v* > 0 are bounded parameters, and 0 < *ε* ≪ 1 is perturbation parameter. And then we get the real normal form of the system (2) after a series of transformations. Two examples are provided to illustrate these results in the last section.

## 2. Preliminary Theorems

In this section, we first consider a normal form system of degree two in ℝ^3^. Then, by a series of transformations we introduce some theorems for the normal form. The reader is referred to [15] for more details about the following content.

Consider the 3-dimensional system

(3) $$\begin{array}{c} \dot{x}=Dx+X_{2}\left(x\right), \end{array}$$

where *X*_2_(*x*) = *O*(|*x*|^2^) is *C* ^*∞*^ in *x* ∈ ℝ^3^, and

(4) $$\begin{array}{c} D=\left(\begin{array}{ccc} 0 & 1 & 0\\ -1 & 0 & 0\\ 0 & 0 & 0 \end{array}\right). \end{array}$$

By adding up the 2-parameter linear part diag⁡(*λ*_1_, *λ*_1_, *λ*_2_)*x* we obtain

(5) $$\begin{array}{c} \dot{x}=D\left(\lambda_{1},\lambda_{2}\right)x+X_{2}\left(x\right), \end{array}$$

where *D*(*λ*_1_, *λ*_2_) = diag⁡(*A*(*λ*_1_), *λ*_2_), with

(6) $$\begin{array}{c} A\left(\lambda_{1}\right)=\left(\begin{array}{cc} \lambda_{1} & 1\\ -1 & \lambda_{1} \end{array}\right). \end{array}$$

It can be verified that (5) has the following real normal form up to order 3 (see [15]):

(7) x˙1=λ1x1+x2+a1x1x3+b1x2x3+(a2x1+b2x2)×(x12+x22)+(a3x1+b3x2)x32+O(|x1,x2,x3|4),x˙2=−x1+λ1x2−b1x1x3+a1x2x3+(−b2x1+a2x2)×(x12+x22)+(−b3x1+a3x2)x32+O(|x1,x2,x3|4),x˙3=λ2x3+c1(x12+x22)+d1x32+c2(x12+x22)x3+d2x33+O(|x1,x2,x3|4).

For convenience, we assume that *a*_1_ *b*_1_ *c*_1_ ≠ 0 as in [15]. By the scaling

(8) $$\begin{array}{c} x_{1}⟶\frac{\sqrt{\left|d_{1}\right|}}{\sqrt{\left|c_{1}\right|}a_{1}}x_{1},\;\;x_{2}⟶\frac{\sqrt{\left|d_{1}\right|}}{\sqrt{\left|c_{1}\right|}a_{1}}x_{2},\;\;x_{3}⟶\frac{1}{a_{1}}x_{3}, \end{array}$$

(7) becomes

(9) x˙1=λ1x1+x2+x1x3+b~1x2x3+(a~2x1+b~2x2)(x12+x22)+(a~3x1+b~3x2)x32+O(|x1,x2,x3|4),x˙2=−x1+λ1x2−b~1x1x3+x2x3+(−b~2x1+a~2x2)×(x12+x22)+(−b~3x1+a~3x2)x32+O(|x1,x2,x3|4),x˙3=λ2x3+c~1(x12+x22)+d~1x32+c~2(x12+x22)x3+d~2x33+O(|x1,x2,x3|4),

where

(10) $$\begin{array}{c} {\tilde{b}}_{1}=\frac{b_{1}}{a_{1}},\;\;{\tilde{a}}_{2}=\frac{\left|d_{1}\right|a_{2}}{\left|c_{1}\right|a_{1}^{2}},\;\;{\tilde{b}}_{2}=\frac{\left|d_{1}\right|b_{2}}{\left|c_{1}\right|a_{1}^{2}},\\ {\tilde{a}}_{3}=\frac{a_{3}}{a_{1}^{2}},\;\;{\tilde{b}}_{3}=\frac{b_{3}}{a_{1}^{2}},\;\;{\tilde{c}}_{1}=\frac{\left|d_{1}\right|}{a_{1}}\mathrm{sgn}\left(c_{1}\right),\\ {\tilde{d}}_{1}=\frac{d_{1}}{a_{1}},\;\;{\tilde{c}}_{2}=\frac{\left|d_{1}\right|c_{2}}{\left|c_{1}\right|a_{1}^{2}},\;\;{\tilde{d}}_{2}=\frac{d_{2}}{a_{1}^{2}}. \end{array}$$

Then, by introducing polar coordinates

(11) $$\begin{array}{c} x_{1}=p\cos\theta ,\;\;x_{2}=-p\sin\theta , \end{array}$$

(9) further becomes

(12) $$\begin{array}{c} \dot{\theta }=1+{\tilde{b}}_{1}x_{3}+{\tilde{b}}_{2}p^{2}+{\tilde{b}}_{3}x_{3}^{2}+p^{-1}S_{1}\left(\theta ,p,x_{3}\right),\\ \dot{p}=\lambda_{1}p+px_{3}+{\tilde{a}}_{2}p^{3}+{\tilde{a}}_{3}px_{3}^{2}+S_{2}\left(\theta ,p,x_{3}\right),\\ {\dot{x}}_{3}=\lambda_{2}x_{3}+{\tilde{c}}_{1}p^{2}+{\tilde{d}}_{1}x_{3}^{2}+{\tilde{c}}_{2}p^{2}x_{3}+{\tilde{d}}_{2}x_{3}^{3}+S_{3}\left(\theta ,p,x_{3}\right), \end{array}$$

where *S*_1_, *S*_2_, and *S*_3_ are 2*π* periodic in *θ*, and *S*_1_, *S*_2_, and *S*_3_ = *O*(|*p*, *x*_3_|^4^). By a further scaling of the form

(13)   p⟶εp,  x3⟶εx3,λ1⟶εδ1,  λ2⟶εδ2,      ε>0,  |δ1|=1,

(12) becomes

(14) $$\begin{array}{c} \dot{\theta }=1+\varepsilon \left[{\tilde{b}}_{1}x_{3}+\varepsilon \left({\tilde{b}}_{2}p^{2}+{\tilde{b}}_{3}x_{3}^{2}\right)\right]+p^{-1}\varepsilon^{3}O\left({\left|p,x_{3}\right|}^{4}\right),\\ \dot{p}=\varepsilon p\left[\delta_{1}+x_{3}+\varepsilon \left({\tilde{a}}_{2}p^{2}+{\tilde{a}}_{3}x_{3}^{2}\right)\right]+O\left(\varepsilon^{3}\right),\\ {\dot{x}}_{3}=\varepsilon \left[\delta_{2}x_{3}+{\tilde{c}}_{1}p^{2}+{\tilde{d}}_{1}x_{3}^{2}+\varepsilon \left({\tilde{c}}_{2}p^{2}x_{3}+{\tilde{d}}_{2}x_{3}^{3}\right)\right]+O\left(\varepsilon^{3}\right). \end{array}$$

We obtain from (14)

(15) $$\begin{array}{c} \frac{dp}{d\theta }=\varepsilon p\left[f_{0}\left(p,x_{3}\right)+\varepsilon f_{1}\left(p,x_{3}\right)+\bar{f}\left(\theta ,p,x_{3},\varepsilon \right)\right],\\ \frac{dx_{3}}{d\theta }=\varepsilon \left[g_{0}\left(p,x_{3}\right)+\varepsilon g_{1}\left(p,x_{3}\right)+\bar{g}\left(\theta ,p,x_{3},\varepsilon \right)\right], \end{array}$$

where

(16) $$\begin{array}{c} f_{0}\left(p,x_{3}\right)=\delta_{1}+x_{3},\\ g_{0}\left(p,x_{3}\right)=\delta_{2}x_{3}+{\tilde{c}}_{1}p^{2}+{\tilde{d}}_{1}x_{3}^{2},\\ f_{1}\left(p,x_{3}\right)=-\delta_{1}{\tilde{b}}_{1}x_{3}+{\tilde{a}}_{2}p^{2}+\left({\tilde{a}}_{3}-{\tilde{b}}_{1}\right)x_{3}^{2},\\ g_{1}\left(p,x_{3}\right)=-{\tilde{b}}_{1}\delta_{2}x_{3}^{2}+\left({\tilde{c}}_{2}-{\tilde{c}}_{1}{\tilde{b}}_{1}\right)p^{2}x_{3}+\left({\tilde{d}}_{2}-{\tilde{b}}_{1}{\tilde{d}}_{1}\right)x_{3}^{3},\\ \bar{f},\bar{g}=O\left(\varepsilon^{2}\right). \end{array}$$

Note that the functions $\bar{f}$ and $\bar{g}$ in (15) are 2*π* periodic in *θ* but may not be well defined at *p* = 0. Thus, we suppose *p* ≫ *ε* > 0 for (15).

The averaging system

(17) $$\begin{array}{c} \frac{dp}{d\theta }=pf_{0}\left(p,x_{3}\right),\\ \frac{dx_{3}}{d\theta }=g_{0}\left(p,x_{3}\right) \end{array}$$

has a singular point (*p*_0_, *s*_0_) on the half plane *p* > 0 if

(18) $$\begin{array}{c} {\tilde{c}}_{1}\left({\tilde{d}}_{1}-\delta_{1}\delta_{2}\right)<0, \end{array}$$

where $p_{0}=\sqrt{(\delta_{1}\delta_{2}-{\tilde{d}}_{1})/{\tilde{c}}_{1}},s_{0}=-\delta_{1}$. By denoting

(19) $$\begin{array}{c} B=\frac{\partial \left(pf_{0},g_{0}\right)}{\partial \left(p,x_{3}\right)}{|}_{\left(p_{0},s_{0}\right)}, \end{array}$$

we obtain $|B|=-2{\tilde{c}}_{1}p_{0}^{2}\neq 0$, and the characteristic polynomial of *B* is $f_{\lambda }(B)=\lambda^{2}-(\delta_{2}-2{\tilde{d}}_{1}\delta_{1})\lambda -2({\tilde{d}}_{1}-\delta_{1}\delta_{2})$. We define

(20) $$\begin{array}{c} \Delta ={\left(\delta_{2}-2{\tilde{d}}_{1}\delta_{1}\right)}^{2}+8\left({\tilde{d}}_{1}-\delta_{1}\delta_{2}\right). \end{array}$$

According to Theorem 4.1.3 in [15], we can obtain the following theorem.

**Theorem 1**

Suppose that (18) holds. Then, (7) has a periodic orbit near the origin for 0 < *ε* ≪ 1. Further, the periodic orbit is stable (resp. unstable) if one (resp. none) of the following conditions holds:

- Δ = 0 and $\delta_{2}-2{\tilde{d}}_{1}\delta_{1}<0$,
- Δ < 0 and $\delta_{2}-2{\tilde{d}}_{1}\delta_{1}\leq 0$,
- Δ > 0, $\delta_{2}-2{\tilde{d}}_{1}\delta_{1}<0$, and ${\tilde{d}}_{1}-\delta_{1}\delta_{2}<0$,

where Δ is given by (20).

Then, by letting *s* = *x*_3_ + *δ*_1_ and *θ* → *ε* ^−1^ *θ* and truncating the terms of order *ε* ^2^, we have from (15)

(21) dpdθ=ps+εp[a~2p2+f2(s)],dsdθ=c~1p2+g2(s)+ε[g3(s)p2+g4(s)],

where

(22) f2(s)=(a~3−b~1)s2+δ1(b~1−2a~3)s+a~3,g2(s)=d~1s2+(δ2−2δ1d~1)s+δ1(δ1d~1−δ2),g3(s)=(c~2−c~1b~1)(s−δ1),g4(s)=(d~2−b~1d~1)(s−δ1)3−δ2b~1(s−δ1)2.

Thus, in order that (21) has a limit cycle, we necessarily suppose

(23) $$\begin{array}{c} \delta_{2}-2\delta_{1}{\tilde{d}}_{1}=\delta \varepsilon ,\;\delta \in \mathbb{R}, \end{array}$$

and ${\tilde{c}}_{1}<0$, ${\tilde{c}}_{1}{\tilde{d}}_{1}>0$, that is,

(24) $$\begin{array}{c} a_{1}c_{1}<0,\;\;c_{1}d_{1}>0. \end{array}$$

This yields ${\tilde{c}}_{1}={\tilde{d}}_{1}<0$, and hence (21) becomes

(25) dpdθ=ps+εp[a~2p2+f2(s)],dsdθ=c~1(p2+s2−1)+ε[g3(s)p2+g¯4(s)+O(ε)],

where

(26) g¯4(s)=(d~2−c~1b~1)s3+δ1(c~1b~1−3d~2)s2+(δ+3d~2+c~1b~1)s−(δ+d~2+c~1b~1)δ1.

For small *ε* > 0, (25) has a focus *A*_*ε*_(*p*(*ε*), *s*(*ε*)) with

(27) p(0)=1,  s(0)=0,  s′(0)=−(a~3+a~2).

We define

(28) δ0=2c~1a~3−3d~2−c~2−2a~2(1−c~1),Δ0=18[3d~2−c~2−2a~2(1−c~1)−2c~1a~3],δ0′=23−2c~1[(2−c~1)(2c~1a~3−3d~2)−(1−c~1)    ×(c~2+2a~2(1−c~1))].

By using the coefficients in (7), we have

(29) δ0=2d1a3a13−3d2a12−d1c1a12[c2+2a2(1−d1a1)],Δ0=18[1a13(3a1d2−2a3d1)  −d1c1a12(c2+2a2(1−d1a1))],δ0′=23a1−2d1[(2−d1a1)(2d1a3a12−3d2a1)      −d1c1a1(1−d1a1)(c2+2a2(1−d1a1))].

Then, in 1997, the following result was obtained in [15].

**Theorem 2**

Suppose that (24) holds and Δ_0_ ≠ 0. Then, for any given *ε*_1_ > 0 there exist an *ε*_0_ > 0 and a *C* ^1^ function *ϕ*_0_(*λ*_1_) = (2*d*_1_ *λ*_1_/*a*_1_) + *δ*_0_ *λ*_1_^2^ + *O*(*λ*_1_^3^) and *ϕ*_1_(*λ*_1_) = (2*d*_1_ *λ*_1_/*a*_1_) + *δ*_0_′*λ*_1_^2^ + *O*(*λ*_1_^3^) such that for 0 < *λ*_1_^2^ + *λ*_2_^2^ < *ε*_0_, (7) has a unique invariant torus near the origin if Δ_0_ *ϕ*_1_(*λ*_1_) − *ε*_1_ *λ*_1_^2^ < Δ_0_ *λ*_2_ < Δ_0_ *ϕ*_0_(*λ*_1_) and has no invariant torus if Δ_0_ *λ*_2_ > Δ_0_ *ϕ*_0_(*λ*_1_). Moreover, the torus, if it exists, is stable (resp. unstable) when Δ_0_ < 0 (resp. >0).

## 3. Normal Form of System (2)

In this section, we consider system (1) in the first octant ℝ_+_^3^, where ℝ_+_ = {*x* ∈ ℝ : *x* > 0}. We now look for the conditions for the existence of positive equilibria of system (1), which is equivalent to find the positive solutions of the following system:

(30) $$\begin{array}{c} \beta_{i}+\overset{3}{\underset{j=1}{\sum }}\alpha_{ij}X_{j}=0,\;i=1,2,3. \end{array}$$

We suppose that there exists at least one positive solution of (30). Without loss of generality, we assume that the positive equilibrium is (1,1, 1). Then, we move it to the origin by doing the change of variables *Y*_*i*_ = *X*_*i*_ − 1, *i* = 1,2, 3. Then, system (1) can be written as

(31) dYidt=(Yi+1)∑j=13αijYj, i‍=1,2,3.

Now, we shall investigate a special form of system (31) with a small parameter; we write the perturbed system as

(32) dYidt=(Yi+1)∑j=13αij(ε)Yj, i=1,2,3.

Denote *M*(*ε*) = (*α*_*ij*_(*ε*))_3×3_, and we suppose *M*(*ε*) is similar to

(33) $$\begin{array}{c} \Psi =\left(\begin{array}{ccc} u\varepsilon & v & 0\\ -v & u\varepsilon & 0\\ 0 & 0 & \varepsilon \end{array}\right),\;\left(u,v\right)=\left(u\left(\varepsilon \right),v\left(\varepsilon \right)\right). \end{array}$$

Then, system (32) can be changed into the system (2) by a linear transformation.

In this section, our task is to change system (2) into the normal form of (7). Making the transformation

(34) $$\begin{array}{c} x_{1}=U,\;\;x_{2}=V,\;\;x_{3}=W,\;\;t⟶\frac{1}{v}t, \end{array}$$

system (2) becomes

(35) x˙1=λ1x1+x2+∑i+j+k=2a~ijkx1ix2jx3k,x˙2=−x1+λ1x2+∑i+j+k=2b~ijkx1ix2jx3k‍,x˙3=λ2x3+∑i+j+k=2c~ijkx1ix2jx3k,

where

(36) $$\begin{array}{c} {\tilde{a}}_{ijk}=\frac{1}{v}a_{ijk},\;\;{\tilde{b}}_{ijk}=\frac{1}{v}b_{ijk},\;\;{\tilde{c}}_{ijk}=\frac{1}{v}c_{ijk},\\ \lambda_{1}=\frac{u}{v}\varepsilon ,\;\;\lambda_{2}=\frac{1}{v}\varepsilon . \end{array}$$

Let

(37) $$\begin{array}{c} T=\left(\begin{array}{ccc} 1 & i & 0\\ i & 1 & 0\\ 0 & 0 & 1 \end{array}\right), \end{array}$$

by changing *y* = *Tx*, where *y* = (*y*_1_, *y*_2_, *y*_3_)^*T*^, *x* = (*x*_1_, *x*_2_, *x*_3_)^*T*^, and system (35) becomes a complex system of the form

(38) $$\begin{array}{c} {\dot{y}}_{1}=\left(\lambda_{1}+i\right)y_{1}+\underset{i+j+k=2}{\sum }a_{ijk}^{*}y_{1}^{i}y_{2}^{j}y_{3}^{k},‍\\ {\dot{y}}_{2}=\left(\lambda_{1}-i\right)y_{2}+\underset{i+j+k=2}{\sum }b_{ijk}^{*}y_{1}^{i}y_{2}^{j}y_{3}^{k},\\ {\dot{y}}_{3}=\lambda_{2}y_{3}+\underset{i+j+k=2}{\sum }c_{ijk}^{*}y_{1}^{i}y_{2}^{j}y_{3}^{k}, \end{array}$$

where

(39) a200∗=14(a~200−a~020+b~110)+14(−a~110+b~200−b~020)i,a020∗=14(a~020−a~200+b~110)+14(−a~110+b~020−b~200)i,a002∗=a~002+b~002i,a110∗=12(b~200+b~020)−12(a~200+a~020)i,a101∗=12(a~101−a~011)+12(b~101−b~011)i,a011∗=12(a~011+b~101)+12(−a~101+b~011)i,b200∗=14(b~200−b~020+a~110)+14(−b~110+a~200−a~020)i,b020∗=14(b~020−b~200+a~110)+14(−b~110+a~020−a~200)i,b002∗=b~002+a~002i,b110∗=12(a~200+a~020)−12(b~200+b~020)i,b101∗=12(b~101−b~011)+12(a~101−a~011)i,b011∗=12(b~011+a~101)+12(−b~101+a~011)i,c200∗=14(a~200−a~020)−14a~110i,c020∗=14(a~020−a~200)−14a~110i,c002∗=a~002,c110∗=−12(a~200+a~020)i,c101∗=12(a~101−a~011),c011∗=12a~011−12a~101i.

By the fundamental theory of normal form [16], we know that system (38) can be converted to the normal form by some transformations. So our following task is to find the transformations and work out the normal form of system (38).

We denote (38) as $\dot{y}=F(y)$, where *F*(0) = 0, and for simplicity, we write the nonlinear part of (38) as Θ(*y*). By doing the following transformation:

(40) $$\begin{array}{c} y=z+P\left(z\right)\equiv h_{1}\left(z\right),\;z\in {\mathbb{R}}^{3}, \end{array}$$

where *P*(*z*) = (*P*_1_(*z*), *P*_2_(*z*), *P*_3_(*z*))^*T*^, which is to be determined, (38) becomes

(41) $$\begin{array}{c} \dot{z}={\left[Dh_{1}\left(z\right)\right]}^{-1}F\left(h_{1}\left(z\right)\right). \end{array}$$

Then, by noting

(42) $$\begin{array}{c} {\left(Dh_{1}\right)}^{-1}=I-DP+{\left(DP\right)}^{2}+O\left({\left|DP\right|}^{3}\right), \end{array}$$

we can get from (41)

(43) z˙i=γizi+γiPi−∑j=13∂Pi∂zjγjzj+Θi(z)+O(|z|3), i=1,2,3,

where *γ*_1_ = *λ*_1_ + *i* and *γ*_2_ = *λ*_1_ − *i*, *γ*_3_ = *λ*_2_. In order to eliminate the quadratic homogeneous polynomial, we need

(44) $$\begin{array}{c} \gamma_{i}P_{i}-\overset{3}{\underset{j=1}{\sum }}\frac{\partial P_{i}}{\partial z_{j}}\gamma_{j}z_{j}=-\Theta_{i}\left(z\right)+O\left({\left|z\right|}^{3}\right),\;i=1,2,3. \end{array}$$

We take *P*_*i*_, *i* = 1,2, 3 as quadratic homogeneous polynomial, having the form

(45) $$\begin{array}{c} P_{i}=l_{i1}z_{1}^{2}+l_{i2}z_{2}^{2}+l_{i3}z_{3}^{2}+l_{i4}z_{1}z_{2}+l_{i5}z_{1}z_{3}+l_{i6}z_{2}z_{3}, \end{array}$$

where *l*_*ik*_, *k* = 1,…, 6, are real undermined coefficients. By inserting (45) into (44) and comparing the coefficients of similar items, we can obtain

(46) $$\begin{array}{c} l_{11}=\frac{a_{200}^{*}}{\gamma_{1}},\;\;l_{12}=\frac{a_{020}^{*}}{2\gamma_{2}-\gamma_{1}},\;\;l_{13}=\frac{a_{002}^{*}}{2\gamma_{3}-\gamma_{1}},\\ l_{14}=\frac{a_{110}^{*}}{\gamma_{2}},\;\;l_{15}=\frac{a_{101}^{*}}{\gamma_{3}},\;\;l_{16}=\frac{a_{200}^{*}}{\gamma_{2}+\gamma_{3}-\gamma_{1}};\\ l_{21}=\frac{b_{200}^{*}}{2\gamma_{1}-\gamma_{2}},\;\;l_{22}=\frac{b_{020}^{*}}{\gamma_{2}},\;\;l_{23}=\frac{b_{002}^{*}}{2\gamma_{3}-\gamma_{2}},\\ l_{24}=\frac{b_{110}^{*}}{\gamma_{1}},\;\;l_{25}=\frac{b_{101}^{*}}{\gamma_{1}+\gamma_{3}-\gamma_{2}},\;\;l_{26}=\frac{a_{200}^{*}}{\gamma_{3}};\\ l_{31}=\frac{c_{200}^{*}}{2\gamma_{1}-\gamma_{3}},\;\;l_{32}=\frac{c_{020}^{*}}{2\gamma_{2}-\gamma_{3}},\;\;l_{33}=\frac{c_{002}^{*}}{\gamma_{3}},\\ l_{34}=\frac{c_{110}^{*}}{\gamma_{1}+\gamma_{2}-\gamma_{3}},\;\;l_{35}=\frac{c_{101}^{*}}{\gamma_{1}},\;\;l_{36}=\frac{c_{200}^{*}}{\gamma_{2}}. \end{array}$$

Note that |*γ*_3_ | = |*λ*_2_ | ≪1, |*γ*_1_ + *γ*_2_ − *γ*_3_ | = |2*λ*_1_ − *λ*_2_ | ≪1. The terms with coefficients *l*_15_, *l*_26_, *l*_33_, and *l*_34_ that appeared above cannot be removed. Those terms are called the resonance terms. Then, we have

(47) P1=l11z12+l12z22+l13z32+l14z1z2+l16z2z3,P2=l21z12+l22z22+l23z32+l24z1z2+l25z1z3,P3=l31z12+l32z22+l35z1z3+l36z2z3,

and system (43) becomes

(48) z˙1=γ1z1+a101∗z1z3+O(|z1,z2,z3|3),z˙2=γ2z2+b011∗z2z3+O(|z1,z2,z3|3),z˙3=γ3z3+c002∗z32+c110∗z1z2+O(|z1,z2,z3|3).

Let *L*(*z*) denote the cubic terms in *z* of (48). Then, from (41) and (42) we have

(49) $$\begin{array}{c} L\left(z\right)=\left(\begin{array}{c} -\overset{3}{\underset{j=1}{\sum }}P_{1j}\gamma_{j}P_{j}\\ -\overset{3}{\underset{j=1}{\sum }}P_{2j}\gamma_{j}P_{j}\\ -\overset{3}{\underset{j=1}{\sum }}P_{3j}\gamma_{j}P_{j} \end{array}\right)+\left(\begin{array}{c} h_{1}\\ h_{2}\\ h_{3} \end{array}\right)+{\left(DP\right)}^{2}\left(\begin{array}{c} \gamma_{1}z_{1}\\ \gamma_{2}z_{2}\\ \gamma_{3}z_{3} \end{array}\right), \end{array}$$

where *P*_*ij*_ = ∂*P*_*i*_/∂*z*_*j*_, *i*, *j* = 1,2, 3,

(50) h1=−P11(a200∗z12+a020∗z22+a002∗z32)−P12(b200∗z12+b020∗z22+b002∗z32)−P13(c200∗z12+c020∗z22+c002∗z32)−P11(a110∗z1z2+a101∗z1z3+a011∗z2z3)−P12(b110∗z1z2+b101∗z1z3+b011∗z2z3)−P13(c110∗z1z2+c101∗z1z3+c011∗z2z3)+2(a200∗z1P1+a020∗z2P2+a002∗z3P3)+a110∗(z1P2+z2P1)+a101∗(z1P3+z3P1)+a011∗(z2P3+z3P2),h2=−P21(a200∗z12+a020∗z22+a002∗z32)−P22(b200∗z12+b020∗z22+b002∗z32)−P23(c200∗z12+c020∗z22+c002∗z32)−P21(a110∗z1z2+a101∗z1z3+a011∗z2z3)−P22(b110∗z1z2+b101∗z1z3+b011∗z2z3)−P23(c110∗z1z2+c101∗z1z3+c011∗z2z3)+2(b200∗z1P1+b020∗z2P2+b002∗z3P3)+b110∗(z1P2+z2P1)+b101∗(z1P3+z3P1)+b011∗(z2P3+z3P2),h3=−P31(a200∗z12+a020∗z22+a002∗z32)−P32(b200∗z12+b020∗z22+b002∗z32)−P33(c200∗z12+c020∗z22+c002∗z32)−P31(a110∗z1z2+a101∗z1z3+a011∗z2z3)−P32(b110∗z1z2+b101∗z1z3+b011∗z2z3)−P33(c110∗z1z2+c101∗z1z3+c011∗z2z3)+2(c200∗z1P1+c020∗z2P2+c002∗z3P3)+c110∗(z1P2+z2P1)+c101∗(z1P3+z3P1)+c011∗(z2P3+z3P2).

By substituting (42) into the above, we obtain

(51) $$\begin{array}{c} L\left(z\right)=\left(\begin{array}{c} e_{11}z_{1}^{3}+e_{12}z_{2}^{3}+e_{13}z_{3}^{3}+e_{14}z_{1}^{2}z_{2}+e_{15}z_{1}^{2}z_{3}+e_{16}z_{1}z_{2}^{2}+e_{17}z_{1}z_{3}^{2}+e_{18}z_{2}^{2}z_{3}+e_{19}z_{2}z_{3}^{2}+e_{10}z_{1}z_{2}z_{3}\\ e_{21}z_{1}^{3}+e_{22}z_{2}^{3}+e_{23}z_{3}^{3}+e_{24}z_{1}^{2}z_{2}+e_{25}z_{1}^{2}z_{3}+e_{26}z_{1}z_{2}^{2}+e_{27}z_{1}z_{3}^{2}+e_{28}z_{2}^{2}z_{3}+e_{29}z_{2}z_{3}^{2}+e_{20}z_{1}z_{2}z_{3}\\ e_{31}z_{1}^{3}+e_{32}z_{2}^{3}+e_{33}z_{3}^{3}+e_{34}z_{1}^{2}z_{2}+e_{35}z_{1}^{2}z_{3}+e_{36}z_{1}z_{2}^{2}+e_{37}z_{1}z_{3}^{2}+e_{38}z_{2}^{2}z_{3}+e_{39}z_{2}z_{3}^{2}+e_{30}z_{1}z_{2}z_{3} \end{array}\right), \end{array}$$

where

(52) e11=a101∗c200∗2γ1−γ3+2a200∗2γ1+a110∗b200∗2γ1−γ2,e12=2a020∗b020∗γ2+a020∗a110∗2γ2−γ1+a011∗c020∗2γ2−γ3,e13=a002∗(a101∗−2c002∗)2γ3−γ1+a011∗b002∗2γ3−γ2,e14=2a020∗b200∗2γ1−γ2+a011∗c200∗2γ1−γ3+a110∗(a200∗+b110∗)γ1+2a200∗a110∗γ2,e15=a110∗b101∗γ1+γ3−γ2+a101∗(c101∗−a200∗)γ1+2a002∗c200∗2γ1−γ3+a011∗b200∗2γ1−γ2,e16=2a020∗b110∗γ1+a110∗(a110∗+b020∗)γ2+a101∗c020∗2γ2−γ3−a011∗c110∗γ2+γ3−γ1+2a200∗a020∗2γ2−γ1,e17=a011∗b101∗γ1+γ3−γ2+2a200∗a002∗2γ3−γ1+2a002∗c101∗γ1+a110∗b002∗2γ3−γ2,e18=a110∗a011∗γ2+γ3−γ1+a020∗(a101∗−2b011∗)2γ2−γ1+a011∗(b020∗+c011∗)γ2+2a002∗c020∗2γ2−γ3,e19=a011∗(a101∗−b011∗−c002∗)γ2+γ3−γ1+2a020∗b002∗2γ3−γ2+2a002∗c011∗γ2+a002∗a110∗2γ3−γ1,e10=2a200∗a011∗γ2+γ3−γ1+a101∗c011∗−a110∗b011∗γ2−2a002∗c110∗2γ3−γ1+a011∗(b110∗+c101∗)γ1+2a020∗b101∗γ1+γ3−γ2;e21=2a200∗b200∗γ1+b200∗b110∗2γ1−γ2+b101∗c200∗2γ1−γ3,e22=a020∗b110∗2γ2−γ1+2b020∗2γ2+b011∗c020∗2γ2−γ3,e23=b002∗(b011∗−2c002∗)2γ3−γ2+a002∗b101∗2γ3−γ1,e24=2b020∗b200∗2γ1−γ2+b011∗c200∗2γ1−γ3+b110∗(a200∗+b110∗)γ1+2b200∗a110∗γ2,e25=b110∗b101∗γ1+γ3−γ2+b101∗(c101∗+a200∗)γ1+2b002∗c200∗2γ1−γ3+b200∗(b011∗−2a101∗)2γ1−γ2,e26=2b020∗b110∗γ1+b110∗(a110∗+b020∗)γ2+b101∗c020∗2γ2−γ3+2b200∗a020∗2γ2−γ1,e27=b101∗(b011∗−a101∗−c002∗)γ1+γ3−γ2+2b200∗a002∗2γ3−γ1+2b002∗c101∗γ1+b110∗b002∗2γ3−γ2,e28=b110∗a011∗γ2+γ3−γ1+a020∗b101∗2γ2−γ1+b011∗(c011∗−b020∗)γ2+2b002∗c020∗2γ2−γ3,e29=a011∗b101∗γ2+γ3−γ1+2b020∗b002∗2γ3−γ2+2b002∗c011∗γ2+a002∗b110∗2γ3−γ1,e20=2b200∗a011∗γ2+γ3−γ1+b101∗(c011∗+a110∗)γ2−2b002∗c110∗2γ3−γ2+b011∗c101∗−b110∗a101∗γ1+2b020∗b101∗γ1+γ3−γ2;e31=c101∗c200∗2γ1−γ3+2a200∗c200∗γ1+c110∗b200∗2γ1−γ2,e32=2c020∗b020∗γ2+a020∗c110∗2γ2−γ1+c011∗c020∗2γ2−γ3,e33=a002∗c101∗2γ3−γ1+c011∗b002∗2γ3−γ2,e34=2c020∗b200∗2γ1−γ2+c011∗c200∗2γ1−γ3+c110∗(a200∗+b110∗−c101∗)γ1+2c200∗a110∗γ2,e35=c110∗b101∗γ1+γ3−γ2+c101∗(c101∗+a200∗)γ1+2c200∗(c002∗−a101∗)2γ1−γ3+c011∗b200∗2γ1−γ2,e36=2c020∗b110∗γ1+c110∗(a110∗+b020∗−c011∗)γ2+c101∗c020∗2γ2−γ3+2c200∗a020∗2γ2−γ1,e37=c011∗b101∗γ1+γ3−γ2+2c200∗a002∗2γ3−γ1+c101∗(c002∗−a101∗)γ1+c110∗b002∗2γ3−γ2,e38=c110∗a011∗γ2+γ3−γ1+a020∗c101∗2γ2−γ1+c011∗(b020∗+c011∗)γ2+2c020∗(c002∗−b011∗)2γ2−γ3,e39=a011∗c101∗γ2+γ3−γ1+2c020∗b002∗2γ3−γ2+c011∗(c002∗−b011∗)γ2+a002∗c110∗2γ3−γ1,e30=2c200∗a011∗γ2+γ3−γ1+c011∗(b110∗+c101∗)γ2+c011∗(b110∗+c101∗)γ1+2c020∗b101∗γ1+γ3−γ2.

We make a further change *z* = *w* + *Q*(*w*) ≡ *h*_2_(*w*), where *Q* = (*Q*_1_, *Q*_2_, *Q*_3_) is homogeneous cubic polynomial, so that (48) becomes

(53) w˙=[Dh2(w)]−1·z˙=(I−DQ+O(|DQ|2))·z˙=(γ1w1+a101∗w1w3γ2w2+b011∗w2w3γ3w3+c002∗w32+c110∗w1w2) +(γ1Q1−∑j=13Q1jγjwjγ2Q2−∑j=13Q2jγjwj‍γ3Q3−∑j=13Q3jγjwj) +L(w)+O(w4),

where *Q*_*ij*_ = ∂*Q*_*i*_/∂*w*_*j*_, *i*, *j* = 1,2, 3 and *L* has the form as before. In order to eliminate some possibly cubic terms, we consider the equations below

(54) $$\begin{array}{c} \gamma_{i}Q_{i}-\overset{3}{\underset{j=1}{\sum }}Q_{ij}\gamma_{j}w_{j}+L_{i}\left(w\right)=0,\;i=1,2,3. \end{array}$$

Suppose that for *i* = 1,2, 3,

(55) Qi=qi1w13+qi2w23+qi3w33+qi4w12w2+qi5w12w3+qi6w1w22+qi7w1w32+qi8w22w3+qi9w2w32+qi0w1w2w3.

By inserting these representations into (54), we can solve as before

(56) Q1=e112γ1w13+e123γ2−γ1w23+e133γ3−γ1w33+e15γ1+γ3w12w3+e162γ2w1w22+e182γ2+γ3−γ1w22w3+e19γ2+2γ3−γ1w2w32+e10γ2+γ3w1w2w3,Q2=e213γ1−γ2w13+e222γ2w23+e233γ3−γ2w33+e242γ1w12w2+e252γ1+γ3−γ2w12w3+e27γ1+2γ3−γ2w1w32+e28γ2+γ3w22w3+e20γ1+γ3w1w2w3,Q3=e313γ1−γ3w13+e323γ2−γ3w23+e342γ1+γ2−γ3w12w2+e352γ1w12w3+e36γ1+2γ2−γ3w1w22+e37γ1+γ3w1w32+e382γ2w22w3+e39γ2+γ3w2w32.

Hence, system (53) becomes now

(57) w˙1=γ1w1+a101∗w1w3+e14w12w2+e17w1w32+O(|w1,w2,w3|4),w˙2=γ2w2+b011∗w2w3+e26w1w22+e29w2w32+O(|w1,w2,w3|4),w˙3=γ3w3+c002∗w32+c110∗w1w2+e33w33+e30w1w2w3+O(|w1,w2,w3|4),

where *w* and all of the coefficients are complex. Finally making the change *w* = *Tx* and then taking the real parts of *x* and the coefficients of all terms of the resulting system, we can get a cubic real normal form of the form (7) with

(58) a1=14(2a~101+b~011−a~011),b1=14(a~011+b~011−2b~101),c1=12(c~200+c~020),d1=c~002,a2=18(λ12+1)[λ1(5a~2002+5b~0202−a~0202−b~2002        +4a~200a~020+4b~200b~020+3b~110        ×(a~200+a~020)+3a~110(b~200+b~020))      −2a~200b~200+2a~020b~020      +a~110(a~200+a~020)−b~110(b~200+b~020)]−λ18(λ12+9)×[(a~020−a~200+b~110)2+(−a~110+b~020−b~200)2]−18[(2λ1−λ2)2+4]×(−2((a~011+b~101)(c~200−c~020)    +(−a~101+b~011)c~110)  +(2λ1−λ2)((−a~101+b~011)(c~200−c~020)          −(a~011+b~101)c~110)),b2=18(λ12+1)×(−λ1(6a~200b~200−6a~020b~020+3b~110(b~200+b~020)     −3a~110(a~200+a~020))+a~2002+3a~0202+3b~2002  +b~0202+4a~200a~020+4b~200b~020−b~110(a~200+a~020)  −a~110(b~200+b~020))38(λ12+9)×((a~020−a~200+b~110)2+(−a~110+b~020−b~200)2)−18[(2λ1−λ2)2+4]×((2λ1−λ2)((a~011+b~101)(c~200−c~020)         +(−a~101+b~011)c~110)  +2((−a~101+b~011)(c~200−c~020)     −(a~011+b~101)c~110)),a3=12[(2λ2−λ1)2+1]×((2λ2−λ1)  ×(a~002(2a~200+b~110)+b~002(2b~020+a~110))  +a~002(2b~020+a~110)−b~002(2a~200+b~110))+c~011(λ2((a~011+b~101)(b~101−b~011)−(−a~101+b~011)      ×(a~101−a~011))    +2((a~011+b~101)(a~101−a~011)       +(−a~101+b~011)(b~101−b~011)))+1λ12+1(λ1a~002+b~002)(a~101−a~011),b3=12[(2λ2−λ1)2+1]×((a~002(2a~020−b~110)+b~002(2b~200−a~110))  −(2λ2−λ1)  ×(a~002(2b~200−a~110)−b~002(2a~020−b~110))) +14(λ22+4)(2((a~011+b~101)(b~101−b~011)         −(−a~101+b~011)(a~101−a~011))        −λ2((a~011+b~101)(a~101−a~011)           +(−a~101+b~011)           ×(b~101−b~011)))+1λ12+1 ×(a~002−λ1b~002)(a~101−a~011),c2=λ12(λ12+1)[c~011(b~200+b~020)      +c~101(a~200+a~020+c~101−c~011)]+λ24(λ22+4)(c~110(a~011+2b~101−b~011)       +(2a~101−b~011−a~011)       ×(c~200−c~020))+12(λ22+4)(c~110(2a~101−b~011−a~011)       −(a~011+2b~101−b~011)       ×(c~200−c~020)),d2=2λ2−λ12[(2λ2−λ1)2+1]×(2a~002c~101−a~002c~011+b~002c~011)+12[(2λ2−λ1)2+1]×(a~002c~011−2b~002c~101+b~002c~011).

Then, by the equations in (36), we finally get the relationship between the coefficients of the system (2) and of the normal form (7).

## 4. Examples

### 4.1. An Example about the Existence of a Limit Cycle in Three-Dimensional Lotka-Volterra Systems

In this section, we construct a concrete example of three-dimensional Lotka-Volterra systems according to Theorem 1. It is shown that this system undergoes nonisolated zero-Hopf bifurcation.

We consider the following three-parameter Lotka-Volterra system in the first octant ℝ_+_^3^. Consider

(59) dxdt=x(−vx+vy+vz−v),dydt=y(−2vx−2vy−vz+5v),dzdt=z2v2(−x(6v3+6v2ε+10v2uε     +6vuε2+3vu2ε2+u2ε3)  −y(−6v3−2v2uε+2vuε2+vu2ε2+u2ε3)  +z(6v3+2v2ε+4v2uε)  +(−6v3+4v2ε+4v2uε+8vuε2    +4vu2ε2+2u2ε3)),

where 0 < *ε* ≪ 1, *v* > 0 and *u* are bounded parameters.

First of all, we need to change the system (59) to the form of system (2) as in [14]. It can be checked that the point (1,1, 1) is zero-Hopf equilibrium of system (59). We do the change of variables *X* = *x* − 1, *Y* = *y* − 1, and *Z* = *z* − 1 to obtain

(60) dXdt=v(1+X)(−X+Y+Z),dYdt=v(1+Y)(−2X−2Y−Z),dZdt=1+Z2v2(−X(6v3+6v2ε+10v2uε+6vuε2      +3vu2ε2+u2ε3)   −Y(−6v3−2v2uε+2vuε2+vu2ε2+u2ε3)   +Z(6v3+2v2ε+4v2uε)).

The Jacobian matrix of system (60) at (0,0, 0) has eigenvalues *ε*, *εu* + *vi* and *εu* − *vi* with *v* > 0. According to [14], in order to obtain the real Jordan normal form of system (60) at the origin, we do the linear transformation

(61) $$\begin{array}{c} \left(\begin{array}{c} U_{1}\\ V_{1}\\ W_{1} \end{array}\right)=\left(\begin{array}{ccc} p_{11} & p_{12} & p_{13}\\ p_{21} & 1 & 0\\ p_{31} & p_{32} & 1 \end{array}\right)\left(\begin{array}{c} X\\ Y\\ Z \end{array}\right), \end{array}$$

where *p*_11_ = −(*uε* ^2^ + 3*vε* + 3*v* *uε* + 5*v* ^2^)/(*v*(*v* + *ε*)), *p*_12_ = −(−*v* ^2^ + *v* *uε* + *vε* + *uε* ^2^)/(*v*(*v* + *ε*)), *p*_13_ = 2*v*/(*v* + *ε*), *p*_21_ = (3*v* + *ε*)/(*v* + *ε*), *p*_31_ = −(6*v* ^2^ + 6*v* *uε* + *u* ^2^ *ε* ^2^)/2*v* ^2^, and *p*_32_ = −*uε*(2*v* + *uε*)/2*v* ^2^. Then, in the new variables (*U*_1_, *V*_1_, and *W*_1_) system (60) becomes

(62) dU1dt=uεU1+vV1+∑i+j+k=2aijkU1iV1jW1k,dV1dt=−vU1+uεV1+∑i+j+k=2bijkU1iV1jW1k,dW1dt=εW1+∑i+j+k=2cijkU1iV1jW1k,

where *a*_*ijk*_, *b*_*ijk*_, and *c*_*ijk*_ have the following expressions:

(63) a011=21v+(−11+90u)ε+O(ε2),a020=3v+(24−17u)ε+O(ε2)a002=−18v−(36u+30)ε+O(ε2),a110=29v+(163+93u)ε+O(ε2),a101=−30v−(60u+72)ε+O(ε2),a200=−9v−(22u+40)ε+O(ε2),b101=−6v+22ε+O(ε2),b011=−12v−(6u+15)ε+O(ε2),b110=−9v−24ε+O(ε2),b200=−6v+8ε+O(ε2),b020=4v+(−223+15u)ε+O(ε2),b002=12ε+O(ε2),c110=36v+(42+102u)ε+O(ε2),c101=−27v−(123+54u)ε+O(ε2),c011=30v+(10+105u)ε+O(ε2),c200=−6v−(60+16u)ε+O(ε2),c002=−18v−(57+36u)ε+O(ε2),c020=(36−32u)ε+O(ε2).

Next, we need to calculate the partial coefficients of the normal form of system (63). We can get ${\tilde{a}}_{ijk}$, ${\tilde{b}}_{ijk}$, and ${\tilde{c}}_{ijk}$ by (36), and then by the formulas of (58) we have

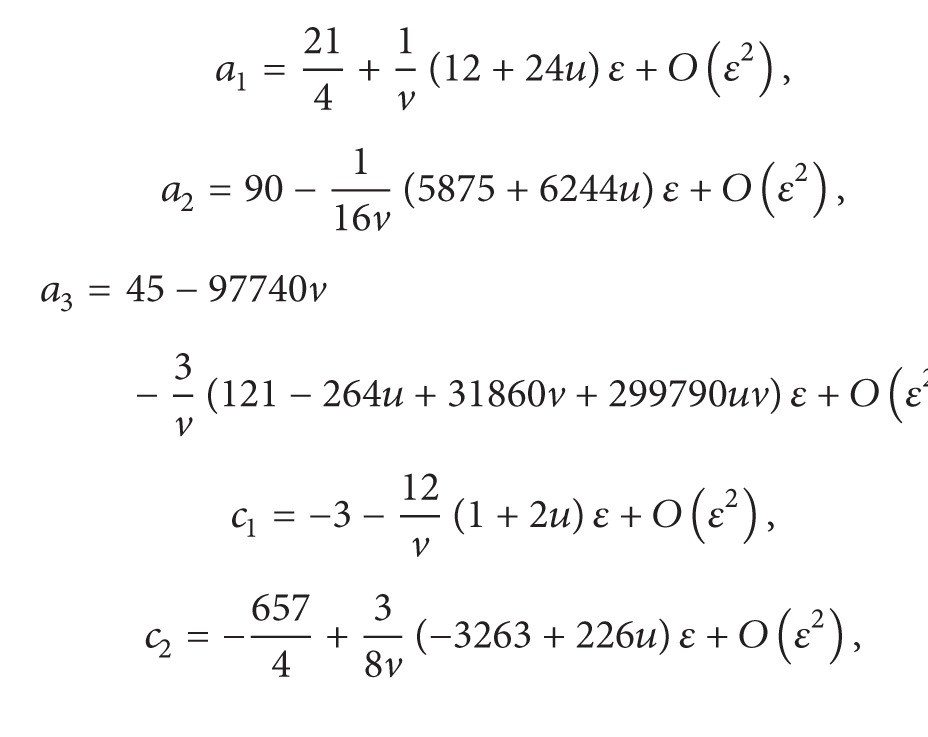
(64)
By Theorem 1, we have the following conclusion.

**Theorem 3**

For any given *ε*_0_ > 0, suppose that −7/(24 + 7*ε*_0_) < *u* < 0, and then for 0 < *ε* ≪ *ε*_0_, (59) has a periodic orbit near the origin, which is unstable.

**Proof**

In this example, it is easy to see that *δ*_1_ = 1, *δ*_2_ = 1/*u*. From (64) and (10) we can get ${\tilde{c}}_{1}={\tilde{d}}_{1}<0$, thus, in order to satisfy (18), we need

(66) $$\begin{array}{c} {\tilde{d}}_{1}-\delta_{1}\delta_{2}=-\frac{24}{7}-\frac{1}{u}+\frac{4}{49}\frac{-37+108u}{v}\varepsilon +O\left(\varepsilon^{2}\right)>0. \end{array}$$

For any given *ε*_0_ > 0, suppose that −7/(24 + 7*ε*_0_) < *u* < 0. It can be checked that ${\tilde{d}}_{1}-\delta_{1}\delta_{2}>0$ for 0 < *ε* ≪ *ε*_0_. Then, by Theorem 1, (59) has a periodic orbit near the origin. Next, we consider the stability of the periodic orbit.

From (64), we can also get

(67) Δ=49+280u+960u249u2−16343×−259−502u+3672u2uv+O(ε2)>0,

when −7/(24 + 7*ε*_0_) < *u* < 0 holds, where Δ is given by (20). So none of the conditions (a), (b), or (c) in Theorem 1 holds; further, we know that the periodic orbit is unstable.

**Remark 4**

From (63), we can find out that system (59) does not satisfy the conditions mentioned in [14]. Thus, we cannot use the results in [14] to study the existence of a limit cycle in (59).

### 4.2. An Example about the Existence of an Invariant Torus

For convenience, we give an example about the existence of an invariant torus in a system, which has the form of (2). We consider the following system in the first octant ℝ_+_^3^:

(68) dU2dt=−524εU2−524V2+3U22−2V22+3W22−15U2V2+2U2W2+5V2W2,dV2dt=524U2−524εV2+9U22−5V22+6W22+U2V2−4V2W2,dW2dt=εW2−4U22+8V22+3W22+510U2V2−7U2W2−5V2W2,

where 0 < *ε* ≪ 1.

According to Section 3, we have

(69) $$\begin{array}{c} a_{1}=6,\\ a_{2}=-\frac{116136}{25}+\frac{1409296}{125}\varepsilon +O\left(\varepsilon^{2}\right),\\ a_{3}=\frac{180576}{25}+\frac{2081376}{125}\varepsilon +O\left(\varepsilon^{2}\right),\\ c_{1}=-\frac{48}{5},\\ c_{2}=\frac{117936}{25}-\frac{3953376}{125}\varepsilon +O\left(\varepsilon^{2}\right),\\ d_{1}=-\frac{72}{5},\\ d_{2}=\frac{11232}{25}+\frac{870048}{125}\varepsilon +O\left(\varepsilon^{2}\right). \end{array}$$

Let *A*_1_ = 1666324684/40625 and *A*_2_ = 1731664/3125. Then, we have the following theorem.

**Theorem 5**

For any given 0 < *ε*_1_ < *A*_1_ − *A*_2_, there exists an *ε*_0_ > 0 such that for 0 < *ε* < *ε*_0_, (68) has a unique invariant torus near the origin, which is unstable.

**Proof**

By (69) and (29), we can obtain

(70) δ0=14892125−1753792375ε−927108063125ε2+O(ε3),Δ0=1325150+2164581875ε+377265312500ε2+O(ε3),δ0′=−2481041625−7012955214625ε−121781598840625ε2+O(ε2).

Thus, for 0 < *ε* ≪ 1, Δ_0_ > 0. Further, we can get

(71) $$\begin{array}{c} \Delta_{0}\phi_{1}\left(\lambda_{1}\right)-\varepsilon_{1}\lambda_{1}^{2}=-\frac{159012}{125}\varepsilon -\left(A_{1}-\varepsilon_{1}\right)\varepsilon^{2}+O\left(\varepsilon^{3}\right),\\ \Delta_{0}\lambda_{2}=-\frac{159012}{125}\varepsilon -A_{2}\varepsilon^{2}+O\left(\varepsilon^{3}\right),\\ \Delta_{0}\phi_{0}\left(\lambda_{1}\right)=-\frac{159012}{125}\varepsilon +\frac{96935282}{3125}\varepsilon^{2}+O\left(\varepsilon^{3}\right), \end{array}$$

where *ϕ*_1_(*λ*_1_) and *ϕ*_0_(*λ*_1_) are defined in Theorem 2, and here *λ*_1_ = *ε* and *λ*_2_ = −(24/5)*ε*. By some easy calculations, we can obtain that for 0 < *ε*_1_ < *A*_1_ − *A*_2_ inequality Δ_0_ *ϕ*_1_(*λ*_1_) − *ε*_1_ *λ*_1_^2^ < Δ_0_ *λ*_2_ < Δ_0_ *ϕ*_0_(*λ*_1_) holds. Thus, by Theorem 2 we can get the result in this theorem.
